# Assessing regional differences in contraceptive discontinuation, failure and switching in Brazil

**DOI:** 10.1186/1742-4755-4-6

**Published:** 2007-07-10

**Authors:** Iúri C Leite, Neeru Gupta

**Affiliations:** 1 Escola Nacional de Saúde Pública, Fundação Oswaldo Cruz, Rio de Janeiro, Brazil; 2 Health Systems and Services, World Health Organization, Geneva, Switzerland

## Abstract

**Background:**

Contraceptive prevalence is relatively high in Brazil (55% among women of reproductive age). However, reversible methods account for less than half of the method mix and widespread differences persist across regions and social groups. This draws attention to the need for monitoring family planning service-related outcomes that might be linked with quality of care. The present study examines the factors associated with method discontinuation, failure and switching among current contraceptive users, with a focus on sub-national assessment.

**Methods:**

Data for the analysis are drawn from the Brazil Demographic and Health Survey, notably the calendar module of reproductive events. Multilevel discrete-time competing risks hazard models are used to estimate the random- and fixed-effects on the probability of a woman making a specific transition after a given duration of contraceptive use.

**Results:**

Contraceptive continuation was found to be highest for the contraceptive pill, the most popular reversible method. Probabilities of abandonment while in need of family planning and of switching to another method were highest for injections. Failure, abandonment and switching were each higher among users in the Northeast region compared to the more prosperous Southeast and South.

**Conclusion:**

Findings point to seemingly important disparities in the availability and quality of family planning and reproductive health care services across regions of the country. Expanding access to a range of contraceptive methods, improving knowledge among health agents of contraceptive technologies and increasing medical supervision of contraceptive practice may be considered key to expanding quality reproductive health care services for all.

## Background

**Figure 1:**
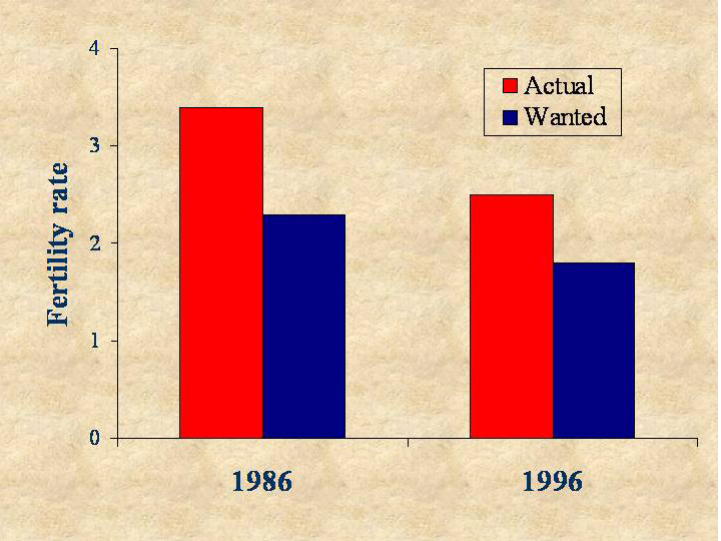
Total and wanted fertility rates, Brazil Demographic and Health Surveys, 1986 and 1996.

Strengthening of reproductive health and family planning services in developing countries is repeatedly highlighted as a priority for reducing maternal mortality and improving maternal and child health. Effective implementation of appropriate services requires an understanding of the factors affecting reproductive outcomes among women at risk and their patterns of behaviour. In Brazil, despite relatively high levels of contraceptive use, there appears to remain a large unmet need for family planning, particularly in the poorer areas of the country. Evidence from the Demographic and Health Survey (DHS) suggests that if all Brazilian women who wanted to limit their fertility were protected by effective contraception, the total fertility rate (TFR) in 1996 would have stood at 1.8 children per woman – one-third lower than the observed rate [1]. This proportion was about the same as that observed ten years earlier, despite the occurrence of rapid fertility decline (Figure 1). Moreover, important differences persist across sub-regions in reproductive health-related behaviours and outcomes.

Differences in availability, accessibility, and acceptability of the range of contraceptive technologies may mean that not all methods are favoured at the same time. Evidence from a number of developing countries reveals that the method mix tends to continuously evolve. It has been estimated that at least half of the women using contraceptives switch methods within a five-year period [2]. A study conducted in six developing countries showed that about one third of women using reversible contraception had stopped using their method within 12 months [3]. Such findings underlie the increasing importance of monitoring trends and determinants of method choice, as family planning and reproductive health programs must adapt to meet users' changing needs and preferences.

In Brazil, reversible methods account for less than half of the method mix [1]. Oral contraceptives are the most commonly used reversible method, the choice of 16% of women of reproductive age. Use of other reversible methods is about 10%. Analyses of DHS results have revealed a number of socio-cultural variables, including women's exposure to the mass media and religion, to have significant influence on contraceptive method choice [4]. Research using multilevel modeling has also pointed to community-level influences on method choice (in particular, significant random effects at the municipal level on adoption of sterilization), likely reflecting influences of the service environment such as presence of hospitals [5]. Brazil does not have an official national family planning program, although in recent years some family planning-related services have been incorporated into the country's maternal and child health program. Evidence has pointed to important constraints in the availability of and access to family planning and reproductive health services, as well as severe deficiencies in quality of care [6].

In order to fully understand women's family planning choices, it is imperative to investigate the components of contraceptive dynamics such as contraceptive discontinuation, failure and switching. Contraceptive failure, resulting in unintended pregnancy, may be due to method failure, user error or provider failure [7]. The impact of contraceptive discontinuation and switching on reproductive outcomes depends to a great extent on both the woman's decision to use another method and the effectiveness of that method. Of particular interest is switching to a less effective method or to no method, which is likely to increase the overall level of fertility. Switching between methods of similar effectiveness may hold less important demographic impact, although any switching may potentially increase the risk of an unintended pregnancy, as women are more likely to experience a method failure in the first months of use when they are not fully familiar with the new method. A study of contraceptive dynamics suggested that at least one-third of the TFR in 15 less developed countries (including Brazil) was associated with either a contraceptive failure or a contraceptive discontinuation for reasons other than a desire to get pregnant [8]. It has been argued that contraceptive continuation rates could be raised substantially by eliminating discontinuation due to non-method- and method-related reasons [9]. The contraceptive continuation rate has been suggested as a useful summary measure of the overall effectiveness of program services in enabling clients to sustain contraceptive use even though they may switch from one method to another [10].

At the same time, Brazilian society has been marked by sharp regional inequalities that have characterised the country since the colonial period [11]. Much attention has been paid to assess the disparities between the poverty-stricken Northeast and more affluent Southeast, the two most populous regions, together comprising about 70% of the total population. For example, in terms of income levels, the proportion of workers earning less than one legal minimum wage is 2.4 times higher in the Northeast (58%) than in the Southeast (24%). A similar tendency can be observed in terms of rates of adult illiteracy (40% versus 9%) [1]. Such differences are seen to hold important implications for demographic and health outcomes. This can be noted through regional variations in the TFR, from a low of 2.1 in the Southeastern state of Rio de Janeiro to a high of 3.1 in the Northeast [1]. Many studies have previously examined the implications of contraceptive continuation on fertility outcomes and on family planning program performance measures at the national level or across countries; however, little research has been conducted at the sub-national level.

The objective of this paper is to analyze the factors associated with contraceptive discontinuation, failure and switching across regions of Brazil, drawing on data from the 1996 DHS. We examine the demographic and socio-cultural influences of contraceptive use dynamics across reversible methods, focusing special attention on the reported reasons for method discontinuation. Identifying the predictors of method failure and discontinuation in the context of rapid and profound changes in reproductive behaviours could draw the attention of policymakers on regional disparities and the difference in quality of reproductive health care provision and eventually assist in improving services.

## Data and methods

The DHS is one of the largest programs collecting quantitative data on reproductive health knowledge, attitudes and practices in the developing world. Surveys are carried out using standardized instruments, methods of training, data collection and data processing [12]. The most recent DHS in Brazil, the 1996 *Pesquisa Nacional sobre Demografia e Saúde*, collected information through personal interviews with 12,612 women aged 15–49, selected through a two-stage random sampling process designed to represent 95% of the country's population at the national and sub-regional levels (some rural areas in the North and Centre-West regions were excluded) [1].

In addition to core questions for measuring basic indicators for population and health program monitoring and evaluation, some DHS surveys include additional modules designed to obtain specialized information on specific topics. The present analysis takes advantage of the "calendar" module of reproductive events. The calendar records detailed information (i.e. month-by-month) about the timing of a number of events–including marital unions, residential mobility, births, and contraceptive use (including method or reason for discontinuation)–occurring in the five calendar years preceding the survey. This retrospective method of measurement makes heavy demands on the memory of respondents, but recall is aided by timing events in relation to one another. Overall, the quality of information obtained through this approach has been evaluated as superior to alternative retrospective data collection techniques for longitudinal information [13,14]. A relatively less-exploited module among the DHS surveys, the calendar has become increasingly important in monitoring contraceptive dynamics and has greatly facilitated researchers' capabilities to conduct analyses of discontinuation and switching in particular [15].

For this study, a discrete-time competing risks hazard model is used to estimate the probability of a woman making a specific transition at a given duration of use. A discrete-time competing risks model is a multinomial logistic model in which the observations are repeated according to the duration of use until the event occurs or is censored. This approach allows incorporation of time-varying covariates as compiled in the calendar (such as woman's age, marital status, and parity at the time of use). Our main objective is to describe the patterns and explain the independent determinants of contraceptive discontinuation, failure and switching among women at risk across the main regions of the country.

Included in the model are all women who were ever sexually active and who initiated use of a reversible method of contraception over the period covered by the calendar. The units of analysis are the episodes of contraceptive use (i.e. continuous use, month-to-month). Observations in the three-month period immediately before the survey are excluded, to reduce bias in the estimation of use-failure rates, given that some women may have been unaware of their very early pregnancy [8]. Likewise excluded are episodes of use that began before the calendar period, as the date of initiation would not have been recorded.

We consider episodes of use of oral contraceptives, injections, condoms, and traditional or natural family planning methods (periodic abstinence and withdrawal). Uses of other modern reversible methods (such as IUD, diaphragm, and spermicides) are excluded, due to the small number of episodes observed in the survey and since it was not considered appropriate to aggregate these methods into a single category as discontinuation and failure rates can vary substantially across methods. Sterilization is also excluded given that discontinuation of this method occurs infrequently.

In examining the patterns of contraceptive use dynamics, four categories were created for the response variable: (i) failure; (ii) abandonment of the method while still in need of family planning; (iii) switching to another reversible method; and (iv) continuing use of the method. Contraceptive failures include any (presumably unintentional) occurrence of a pregnancy while using the method. Episodes where the woman reported having discontinued use for non-method-related reasons–such as a desire to get pregnant, marital separation, or infrequent sexual intercourse–were included under the fourth category, as they were not considered to have ended while in need of family planning. Note that these categories should be interpreted as approximate, given that self-reported reasons for contraceptive discontinuation may be somewhat unreliable [16].

A number of episode-specific and woman-specific variables were included in the model as potential confounding factors, including contraceptive intention and duration of use as well as woman's age, marital status, number of living children, education, ethnicity, place of residence (according to the residential history in the calendar), and mass media exposure (as assessed through television viewing habits). These covariates have been previously considered relevant to assess mechanisms that influence contraceptive use, method choice and/or discontinuation in Brazil and other developing countries [2-4,15-17]. In particular, television programming, notably the highly popular soap operas (telenovelas), has been credited with playing a substantial role in promoting ideological change in Brazil with respect to reproductive behaviours by portraying lifestyles that favour smaller families [18]. Moreover, in the Brazilian context, particular attention is paid to differentials across sub-regions.

Of further substantive and methodological interest, our study uses a multilevel approach. Standard regression models assume that observations are independent. However, given the hierarchically nested structure of the data being used here, multilevel modeling becomes necessary to allow for controlling for any unobserved correlation between observations within hierarchical levels. At the first level, in modeling women's episodes of contraceptive use, an individual may contribute more than one segment of use to the sample. At the second level, the DHS sampling scheme entails selection of households and individuals within enumeration clusters [19]. Individuals from the same sampling cluster are considered likely to exhibit similar demographic and behavioural characteristics (because of a variety of unmeasured and unmeasurable factors) compared to those selected from different clusters. The multilevel model is thus used to compensate for assumed intra-woman and intra-cluster dependence of observations. Moreover, a cluster can be considered a proxy for neighbourhood or community, and reflects local service environment as well as local "culture". It has been argued that women in the same community often talk to each other and, therefore, are more likely to exhibit similar behaviours regarding contraceptive use [20].

The multilevel discrete-time competing risks model is used to assess regional disparities in contraceptive failure, abandonment and switching, conditioned for the set of fixed- and random-effects. The formulation of the model is as follows:

ln⁡(λrtijkλ4tijk)=αrt+β′rxtijk+urjk+vrk,r=1,2,3
 MathType@MTEF@5@5@+=feaafiart1ev1aaatCvAUfKttLearuWrP9MDH5MBPbIqV92AaeXatLxBI9gBaebbnrfifHhDYfgasaacH8akY=wiFfYdH8Gipec8Eeeu0xXdbba9frFj0=OqFfea0dXdd9vqai=hGuQ8kuc9pgc9s8qqaq=dirpe0xb9q8qiLsFr0=vr0=vr0dc8meaabaqaciaacaGaaeqabaqabeGadaaakeaafaqabeqacaaabaGagiiBaWMaeiOBa4MaeiikaGYaaSaaaeaaiiGacqWF7oaBdaWgaaWcbaGaemOCaiNaemiDaqNaemyAaKMaemOAaOMaem4AaSgabeaaaOqaaiab=T7aSnaaBaaaleaacqaI0aancqWG0baDcqWGPbqAcqWGQbGAcqWGRbWAaeqaaaaakiabcMcaPiabg2da9iab=f7aHnaaBaaaleaacqWGYbGCcqWG0baDaeqaaOGaey4kaSIaf8NSdiMbauaadaWgaaWcbaGaemOCaihabeaakiabdIha4naaBaaaleaacqWG0baDcqWGPbqAcqWGQbGAcqWGRbWAaeqaaOGaey4kaSIaemyDau3aaSbaaSqaaiabdkhaYjabdQgaQjabdUgaRbqabaGccqGHRaWkcqWG2bGDdaWgaaWcbaGaemOCaiNaem4AaSgabeaakiabcYcaSaqaaiabdkhaYjabg2da9iabigdaXiabcYcaSiabikdaYiabcYcaSiabiodaZaaaaaa@6798@

where *λ*_*rtijk *_is referred to as the hazard of a transition of type *r *at time *t *for the use interval *i *of woman *j *from cluster *k*. The baseline hazard is represented by *α*_*rt*_, a function of time. *β*_*r *_is the vector of parameters for transition *r*, with *x*_*tijk *_the associated set of covariates (the same for each of the three types of contrasts against continuation of method use). The estimators *u*_*rjk *_and *v*_*rk *_measure the random variations at the woman and cluster levels respectively. They are assumed to be mutually independent and normally distributed with mean zero and variances σrjk2
 MathType@MTEF@5@5@+=feaafiart1ev1aaatCvAUfKttLearuWrP9MDH5MBPbIqV92AaeXatLxBI9gBaebbnrfifHhDYfgasaacH8akY=wiFfYdH8Gipec8Eeeu0xXdbba9frFj0=OqFfea0dXdd9vqai=hGuQ8kuc9pgc9s8qqaq=dirpe0xb9q8qiLsFr0=vr0=vr0dc8meaabaqaciaacaGaaeqabaqabeGadaaakeaaiiGacqWFdpWCdaqhaaWcbaGaemOCaiNaemOAaOMaem4AaSgabaGaeGOmaidaaaaa@33BE@ and σrk2
 MathType@MTEF@5@5@+=feaafiart1ev1aaatCvAUfKttLearuWrP9MDH5MBPbIqV92AaeXatLxBI9gBaebbnrfifHhDYfgasaacH8akY=wiFfYdH8Gipec8Eeeu0xXdbba9frFj0=OqFfea0dXdd9vqai=hGuQ8kuc9pgc9s8qqaq=dirpe0xb9q8qiLsFr0=vr0=vr0dc8meaabaqaciaacaGaaeqabaqabeGadaaakeaaiiGacqWFdpWCdaqhaaWcbaGaemOCaiNaem4AaSgabaGaeGOmaidaaaaa@3261@ respectively.

The final sample for the study consisted of 6,027 episodes of contraceptive use. The analysis was carried out using the *MLwiN *statistical software program [21]. In order to facilitate interpretation, the results from the multilevel competing risk hazard model were applied to estimate twelve-month cumulative probabilities of contraceptive discontinuation, using the multiple classification analysis (MCA) table [22]. First, conditional probabilities of method discontinuation were calculated for each month. In order to calculate the effect of a specific covariate on the cumulative probability, the others were held at their mean.

## Results

### Descriptive analysis

As seen in Table 1, findings from the Brazil DHS characterize a population that is essentially urban (82%), relatively educated (62% with at least some secondary schooling), and highly exposed to modern mass media communication (89% watching television on a weekly basis). Considering these same variables as indicators of higher development status, it may be ascertained that the Southeast and Southern regions are the most developed regions of the country while the Northeast is the least developed.

**Table 1:** Percentage distribution of women aged 15–49 according to selected background characteristics, by region, Brazil, 1996.

	**Region**					
	**North**	**Northeast**	**Southeast**	**South**	**Centre-West**	**National**
**Age group**						
15–19	23	21	19	17	17	**20**
20–24	18	17	14	13	16	**15**
25–29	15	16	15	15	18	**15**
30–34	14	14	16	16	15	**15**
35–39	13	13	14	15	14	**14**
40–44	11	10	12	14	11	**12**
45–49	6	9	10	10	9	**9**
**Marital status**						
Married/living together	55	58	60	66	63	**60**
Not in union	45	42	40	34	37	**40**
**Number of children**						
0	34	35	34	30	29	**33**
1	15	13	17	19	15	**16**
2	15	15	20	23	22	**19**
3+	36	37	29	28	34	**32**
**Ethnicity**						
White	18	26	49	68	42	**44**
Other	82	74	51	32	58	**56**
**Educational attainment**						
No schooling	4	10	3	3	7	**5**
Primary	27	39	30	31	32	**33**
Secondary	64	47	59	58	54	**55**
Higher	5	4	8	8	7	**7**
**Mass media exposure**						
Watches TV regularly (every week)	89	81	92	92	87	**89**
Does not watch TV	11	19	8	8	13	**11**
**Place of residence**						
Rural	3	30	11	23	16	**18**
Urban	97	70	89	77	84	**82**

**Total**	**100**	**100**	**100**	**100**	**100**	**100**

Source: Demographic and Health Survey (N = 12,612 women).

Note: Characteristics refer to those reported at the time of the survey. Some rural areas of the North and Centre-West regions excluded from the sampling frame.

At the time of the survey, 55% of all women of reproductive age, and 77% of married women, were currently using some method of contraception. Of these, over half were relying on either female or male sterilization (Table 2). Among users of reversible methods, the majority used oral contraceptives, followed by condoms. In terms of regional patterns, a relatively higher sterilization rate was observed in the Northeast versus greater use of oral contraceptives in the Southeast and the South.

**Table 2:** Percentage distribution of women aged 15–49 currently using contraception according to method used, by region, Brazil, 1996 DHS.

	**Region**					
	**North**	**Northeast**	**Southeast**	**South**	**Centre-West**	**National**
Female sterilization	65	63	45	33	66	**49**
Pill	15	18	30	44	22	**28**
Condoms	8	6	9	8	4	**8**
Male sterilization	0	1	5	3	2	**3**
Injections	6	2	2	1	1	**2**
Other modern	0	1	2	2	1	**2**
Withdrawal	2	4	4	5	2	**4**
Periodic abstinence	3	4	3	4	2	**4**
Folk methods	1	1	0	0	0	**0**

**Total**	**100**	**100**	**100**	**100**	**100**	**100**

Contraceptive discontinuation rates among the most common reversible methods by self-reported reason for discontinuation are presented for the national level in Table 3. Preliminary analysis of findings from the calendar reveals an overall discontinuation rate of 43% for the five-year period before the survey. The rate was lowest for users of oral contraceptives and highest for users of injections. Fewer than 4% of women cited a desire to become pregnant as the reason for having ended an episode of use. Failure rates were higher with traditional methods, while concerns over side effects were more widely reported among users of hormonal methods (injections and oral contraceptives).

**Table 3:** Percent of women aged 15–49 discontinuing a contraceptive method within 12 months after the start of use, by reason for discontinuation, Brazil, 1996 DHS.

**Reason for discontinuation**	**Contraceptive method**					**Total**
	Pill	Condoms	Injections	Periodic abstinence	Withdrawal	
Method failure	4.8	5.1	4.7	17.0	15.7	**5.9**
To become pregnant	5.0	3.7	4.5	2.9	4.3	**3.7**
Side effects, health	11.8	3.6	27.4	1.5	0.6	**7.7**
All other reasons	23.3	47.7	27.1	35.8	41.6	**26.1**

**All reasons**	**44.8**	**60.0**	**63.7**	**57.1**	**62.2**	**43.4**

Source: MEASURE DHS StatCompiler: .

Note: Based on 5 years of calendar data.

### Results from the competing risks hazard model

The estimated coefficients from the risks hazard model for the influences of selected episode- and woman-level variables on contraceptive use dynamics are presented in Table 4. Twelve-month cumulative probabilities of contraceptive failure, abandonment and switching, as derived from these coefficients, can be found in Table 5.

**Table 4:** Estimated coefficients and standard errors from the multilevel competing risks hazard model for the effects on contraceptive failure, abandonment and switching in the five years preceding the survey.

	**Failure**		**Abandonment**		**Switching**	
	***Coefficient***	***S.E.***	***Coefficient***	***S.E.***	***Coefficient***	***S.E.***

**Constant**	-3.050	0.210	-2.772	0.261	-3.994	0.252

**Method**						
Pill	-1.447*	0.092	1.099*	0.170	-0.787*	0.104
Condoms	-0.928*	0.128	0.687*	0.202	0.227	0.117
Injections	-1.083*	0.225	1.600*	0.233	0.354*	0.165
Traditional (ref)	0.000	-	0.000	-	0.000	-
**Duration of use (months)**						
1–3	0.047	0.121	-0.153	0.090	0.338*	0.087
4–6	0.316*	0.120	-0.202*	0.095	0.393*	0.090
7–12	0.162	0.112	-0.304*	0.086	0.068	0.087
13–18	0.022	0.128	-0.105	0.088	0.025	0.095
> 18 (ref)	0.000	-	0.000	-	0.000	-
**Contraceptive intention**						
Spacing (ref)	0.000	-	0.000	-	0.149	0.104
Limiting	-0.311*	0.102	-0.217	0.114	0.000	-
**Region**						
North	-0.452*	0.168	-0.038	0.166	-0.255	0.174
Northeast (ref)	0.000	-	0.000	-	0.000	-
Southeast	-0.260*	0.108	-0.888*	0.133	-0.403*	0.123
South	-0.533*	0.139	-1.349*	0.164	-0.883*	0.151
Centre-West	-0.235	0.137	-0.640*	0.163	-0.626*	0.164
**Age**						
≤ 19	0.147	0.117	0.246*	0.105	0.137	0.107
20–24 (ref)	0.000	-	0.000	-	0.000	-
25–29	-0.234*	0.110	-0.068	0.112	0.069	0.099
30–34	-0.145	0.136	0.295*	0.146	0.128	0.129
35+	-0.827*	0.178	0.450*	0.183	-0.145	0.164
**Marital status**						
Married/living together	0.450*	0.123	0.039	0.110	0.274*	0.110
Other (ref)	0.000	-	0.000	-	0.000	-
**N° of living children**						
0 (ref)	0.000	-	0.000	-	0.000	-
1	-0.275*	0.124	-0.343*	0.121	-0.160	0.119
2	-0.186	0.149	-0.485*	0.162	-0.318*	0.153
3+	0.044	0.179	-0.645*	0.206	-0.425*	0.195
**Ethnicity**						
White	-0.202*	0.086	-0.129	0.103	0.293*	0.090
Other (ref)	0.000	-	0.000	-	0.000	-
**Years of schooling**						
0–3 (ref)	0.000	-	0.000	-	0.000	-
4–8	-0.084	0.110	-0.373*	0.132	0.210	0.138
9–11	-0.385*	0.133	-0.815*	0.160	0.147	0.153
12+	-0.618*	0.208	-1.157*	0.248	0.306	0.195
**Mass media exposure**						
Watches TV regularly	-0.049	0.134	-0.628*	0.157	0.358	0.189
Does not watch TV (ref)	0.000	-	0.000	-	0.000	-
**Place of residence**						
Rural (ref)	0.000	-	0.000	-	0.000	-
Urban	-0.033	0.173	-0.434	0.240	0.108	0.182
**Random effects estimators**						
Cluster level	0.007	0.058	0.010	0.081	0.196*	0.077
Woman level	0.155	0.102	3.260*	0.178	2.115*	0.136

Source: 1996 Demographic and Health Survey (N = 80,407 calendar observations). ref = Reference category. *p < 0.05

Note: Episodes of use of reversible contraceptive methods that began before the five-year calendar period or of sterilization use are not included.

**Table 5:** Twelve-month cumulative probabilities of contraceptive failure, abandonment and switching according to selected episode-level and woman-level variables.

	**Failure**	**Abandonment**	**Switching**	**Continuation**
**Method**				
Pill	0.0647	0.1341	0.1607	**0.6404**
Condoms	0.0910	0.0750	0.3761	**0.4578**
Injections	0.0702	0.1694	0.3881	**0.3722**
Traditional methods	0.2272	0.0373	0.2962	**0.4394**
**Contraceptive intention**				
Spacing	0.0724	0.0912	0.2364	**0.6000**
Limiting	0.0979	0.1123	0.2020	**0.5877**
**Region**				
North	0.0661	0.1696	0.2293	**0.5349**
Northeast	0.0965	0.1642	0.2764	**0.4628**
Southeast	0.0868	0.0782	0.2129	**0.6221**
South	0.0721	0.0536	0.1428	**0.7314**
Centre-West	0.0901	0.1014	0.1724	**0.6360**
**Age**				
≤ 19	0.1112	0.1129	0.2270	**0.5489**
20–24	0.1006	0.0923	0.2067	**0.6004**
25–29	0.0802	0.0869	0.2230	**0.6100**
30–34	0.0843	0.1204	0.2283	**0.5694**
35+	0.0449	0.1476	0.1822	**0.6253**
**Marital status**				
Married/living together	0.0957	0.1027	0.2273	**0.5743**
Other	0.0650	0.1049	0.1831	**0.6471**
**N° of living children**				
0	0.0928	0.1352	0.2464	**0.5255**
1	0.0755	0.1023	0.2235	**0.5987**
2	0.0847	0.0911	0.1956	**0.6286**
3+	0.1075	0.0782	0.1771	**0.6372**
**Ethnicity**				
White	0.0767	0.0951	0.2494	**0.5789**
Other	0.0960	0.1106	0.1902	**0.6032**
**Years of schooling**				
0–3	0.1022	0.1644	0.1772	**0.5562**
4–8	0.0951	0.1144	0.2208	**0.5696**
9–11	0.0742	0.0774	0.2177	**0.6308**
12+	0.0587	0.0549	0.2550	**0.6314**
**Mass media exposure**				
Watches TV regularly	0.0865	0.0985	0.2214	**0.5937**
Does not watch TV	0.0895	0.1820	0.1527	**0.5757**
**Global**	0.0869	0.1034	0.2155	**0.5942**

Source: 1996 Demographic and Health Survey (N = 6027 episodes of use).

Note: Episodes of use of reversible contraceptive methods that began before the five-year calendar period or of sterilization use are not included.

The cumulative probability of failure was highest for episodes of use of traditional methods (0.23). Probabilities of abandonment (presumably while in need of family planning) and of method switching were highest for injections. In contrast, the probability of continuation was highest for the contraceptive pill (0.64), the most widely used reversible method overall. Continuation was also high for condoms, albeit at a lower measure than for the pill.

After controlling for the method type and other potentially confounding factors, the probabilities of failure, abandonment and switching were higher in the Northeast region compared to the Southeast and the South, a pattern that was statistically significant (p < 0.05).

The probability of failure was essentially inversely associated with the woman's age at the start of the episode of use, as well as with her educational attainment. Abandonment was more likely among adolescents (19 years and under) as well as older users (30 years and over) compared to users in their twenties. Abandonment was positively correlated with the number of living children at the start of the episode, and inversely associated with educational attainment. Method switching was more common among married users than their unmarried counterparts.

No discernible differences were found according to urban/rural residence (Table 4), a result consistent with analytical findings for contraceptive discontinuation from other countries [23]. At the same time, significant cluster-level random variations were found with respect to contraceptive switching, pointing to additional unmeasured contextual influences that may increase or decrease the probability of a woman changing her method of choice. Such effects may be related to peer influences or proximity of family planning service delivery points.

## Discussion

Over 90% of governments around the world provide either direct or indirect support for contraceptive methods, including that of Brazil [24]. While Brazil does not have an official family planning program, certain related services have been incorporated into the national maternal and child health program, in recognition of the right of individuals and couples to access family planning and reproductive health information and supplies. It is being increasingly recognized that measures for the monitoring and evaluation of family planning service efforts need to go beyond their impact on fertility. In countries where contraceptive prevalence is relatively high, services aiming to reduce the number of unintended pregnancies must pay special interest to the needs of current contraceptive users. Increased attention to quality of care has heightened attention on outcomes that might be associated with the quality of family planning services, notably contraceptive discontinuation and switching [8].

This paper examined regional patterns of contraceptive discontinuation, failure and switching for reversible methods in Brazil, drawing on data from the DHS calendar. Given both the larger size of the Brazilian survey sample (over 12,000 women), as well as the relatively high overall contraceptive prevalence rate, the study offered a valuable opportunity for monitoring of patterns in discontinuation and switching at the sub-national level. In the analysis of discontinuation, particular attention was paid to the reasons for stopping use and differentiating method failure from abandonment while in need for family planning. Multilevel competing risks hazard models served to assess the random- and fixed-effects on contraceptive dynamics.

In summary, the probability of continuation was found to be highest for oral contraceptives. Encouragingly in the face of the HIV/AIDS epidemic, continuation was also high for condoms. As could be expected, the probability of failure was highest with respect to traditional methods. Greater likelihoods of abandonment and switching were found for injections compared to other modern and traditional methods, echoing research results from a number of Latin American countries and reinforcing suggestions that family planning service managers examine more closely the delivery of injectables [8].

After controlling for episode- and individual-specific factors, the probabilities of contraceptive failure, abandonment and switching were each found to be significantly higher in the Northeast compared to the more developed Southeast and South, pointing to seemingly important disparities in the availability and quality of family planning and reproductive health care services across regions of the country.

Moreover, failure for all methods combined was highest among adolescent and less educated users, likely related to higher rates of user error. Research in Brazil and other Latin American countries has reported that women of lower educational attainment, a characteristic considered as proxy for socio-economic status, were less likely to adopt sterilization for contraceptive purposes [25]. Such patterns could partially be a reflection of poor outreach and follow-up of family planning services towards disadvantaged social groups.

An important venue for further research would thus be to examine, for example, effects of the proximity and quality of local service delivery points. As such, one potential future approach could be to merge independently collected data on municipal-level variables for availability and accessibility of health care resources with the DHS individual data.

Many previous studies of contraceptive discontinuation and/or switching have focused only on reports from married women [3,8,9,16]. Often this was due to the nature of the available data, as some DHS countries limited sample coverage to ever-married women. The present analysis took advantage of available calendar data for all women of reproductive age, in a context of widespread sexual activity and contraceptive use regardless of marital status. While the effect of marital status on the probability of contraceptive abandonment was small, method switching was significantly less common among users who were unmarried at the start of the episode compared to married users. Such findings point to the need for further research on contraceptive use dynamics among unmarried women.

A limitation to this analysis may have been a failure to adequately investigate possible biases due to correlation between the observed variables and errors in the model. A recent study in Indonesia using multiprocess models showed that the contraceptive continuation rate is dependent on the contraceptive choice itself, when applied to the effect of choice for IUD and implants [16]. The potential consequences remain uncertain in the context of Brazil, where the use of reversible clinical methods is nonetheless very low, suggesting an interesting path for future study.

Lastly, the data used here were obtained during the course of fieldwork conducted in 1996, the last survey conducted in Brazil under the auspices of the DHS program. The availability of updated information drawing on results from a more recent survey, with a calendar module to allow for comparative analysis, would be essential to assess any changes in trends over the last decade.

## Competing interests

The authors declare that they have no competing interests.

## Authors' contributions

Both authors contributed to the study design. IC Leite performed the data manipulation and statistical programming. Both authors contributed to the data analysis as well as drafting of the manuscript, and have read and approved the final version.
